# Sticky knowledge: A possible model for investigating implementation in healthcare contexts

**DOI:** 10.1186/1748-5908-2-44

**Published:** 2007-12-20

**Authors:** Glyn Elwyn, Mark Taubert, Jenny Kowalczuk

**Affiliations:** 1 Department of Primary Care and Public Health, School of Medicine, Cardiff University, Neuadd Meirionydd, Heath Park, Cardiff, UK; 2 Modus Consulting, PO Box 24:7, Cardiff, UK

## Abstract

**Background:**

In health care, a well recognized gap exists between what we know should be done based on accumulated evidence and what we actually do in practice. A body of empirical literature shows organizations, like individuals, are difficult to change. In the business literature, knowledge management and transfer has become an established area of theory and practice, whilst in healthcare it is only starting to establish a firm footing. Knowledge has become a business resource, and knowledge management theorists and practitioners have examined how knowledge moves in organisations, how it is shared, and how the return on knowledge capital can be maximised to create competitive advantage. New models are being considered, and we wanted to explore the applicability of one of these conceptual models to the implementation of evidence-based practice in healthcare systems.

**Methods:**

The application of a conceptual model called sticky knowledge, based on an integration of communication theory and knowledge transfer milestones, into a scenario of attempting knowledge transfer in primary care.

**Results:**

We describe Szulanski's model, the empirical work he conducted, and illustrate its potential applicability with a hypothetical healthcare example based on improving palliative care services. We follow a doctor through two different posts and analyse aspects of knowledge transfer in different primary care settings. The factors included in the sticky knowledge model include: causal ambiguity, unproven knowledge, motivation of source, credibility of source, recipient motivation, recipient absorptive capacity, recipient retentive capacity, barren organisational context, and arduous relationship between source and recipient. We found that we could apply all these factors to the difficulty of implementing new knowledge into practice in primary care settings.

**Discussion:**

Szulanski argues that knowledge factors play a greater role in the success or failure of a knowledge transfer than has been suspected, and we consider that this conjecture requires further empirical work in healthcare settings.

## Background

Why is it so difficult to spread good practice in organisations? This is an important question for health services needing to improve quality and reduce risk. Transferring best practice is slow, costly, and prone to failure across all industries and public services. Problems associated with implementing change form a vast body of literature across many disciplines [1], but despite this growing body of work, answers remain elusive, and no approach seems substantially better than another [2]. Compounding this, little high-quality empirical evidence can be found to support different approaches [3]. Existing evidence, when available, is hard to compare, based on different disciplinary perspectives and time frames [1].

Recent reviews focusing on how to implement change call attention to the process of knowledge transfer [2-4]. While a large amount of empirical work has extended our knowledge and evidence base for good practice, less has been accomplished on how to implement it [2]. There are huge gaps between what we know and what we do, and these knowing-doing gaps have many consequences [2]. Variations in clinical practice are ubiquitous. For instance, levels of hypertension treatment and control have been noted to vary considerably between Europe and North America [5], and while awareness and familiarity with British Hypertension Society guidelines within the UK is generally high, their actual implementation is inadequate [6].

Numerous lines of work on diffusion and knowledge management exist. Roger's work on the diffusion of innovation is a widely recognised starting point [7], but there are many others who have written about knowledge creation, notably Nonaka [8], about knowledge management [9], the social life of information [10], and on how organisations make sense of information [11]. However, recent work in the field of strategic management has examined the difficulty of spreading innovation [12], and the problem of transferring of best practice from one location to another [13]. This article focuses on one recent approach to this difficulty and considers its application to a health care context. The approach suggests that many difficulties occur because knowledge is sticky and difficult to move. This concept is novel for the health sector and requires discussion. This article examines the concept of sticky knowledge and how it might help us bridge the gap between clinical knowledge and clinical practice.

## Methods

This article is a summary of one author's theoretical construct and empirical work, which has been applied to hypothetical scenarios in primary care, in order to illustrate the potential utility of the approach. It is based on a reading of Szulanski's monograph, where he provides the results of a doctorate conducted at INSEAD, management school, Paris [13]. His empirical work was composed of a cross-sectional survey of intra-firm knowledge transfer that involved 122 transfers of 38 practices in eight global companies, and from the data he developed a conceptual model of knowledge stickiness that we recognised as having good fit and relevance to health care settings. In order to apply the work, we chose to work as a small group to apply the concept of sticky knowledge to a difficult knowledge transfer we had personally experienced in practice.

### Knowledge management and sticky knowledge

Knowledge, and how well it is managed, is recognised as a key to profitability in the new world order of the knowledge economy [14]. Developing competence in managing knowledge is considered essential in establishing competitive market advantage. Drucker stated that the most valuable assets of a 20^th ^Century company were its production equipment, but that the most valuable asset of a 21^st ^Century institution "will be its knowledge workers and their productivity" [15]. There is a growing realisation in health services that knowledge is both unevenly distributed and unequally adopted [16], and just as in business, this heterogeneity is costly, inefficient, and carries a human cost in excess morbidity and mortality [17].

Szulanski, working in the field of strategic management, investigated the factors that make knowledge sticky and how they impact on the process of knowledge transfer [13]. He considered the question why are best practices so difficult to transfer and why do so many attempts at transfer fail? Essentially, knowledge, concepts, and guidelines that are considered sticky are difficult to move from one workplace to another. If they work well in one place, then why can't they work well somewhere else? Or, in a healthcare setting, why does one family practice find it easy to set up, kick-start, successfully implement, and reap the rewards of a clear treatment protocol and another doesn't?

### Using Knowledge effectively in clinical practice

In a clinical practice setting, creativity and effective management in the right environment can lead to success. We will follow a hypothetical doctor through her first two years of working in a generalist family medicine context. At each stage we follow her attempts to implement a recognized framework for providing gold-standard care for terminally ill patients in her new organization [18].

In the case example (see Table 1 Case Study, Year One), the doctor in training has managed to implement a new system in her working environment with excellent results. Let us assume that the Gold Standards Framework for Palliative Care represents best practice. In order to highlight Szulanski's concepts, we will look at a further example to illustrate how the knowledge of successfully implementing this framework metaphorically sticks like chewing-gum to her first working environment as the physician (Kate) tries to replicate the knowledge in her next place of work. Before we do this, it is important to learn about knowledge transfer milestones.

**Table 1:** Case Study, Year 1. Implementing best practice in a receptive environment

Kate is starting out as a family doctor in a rural practice and is undertaking her training year. As part of this vocational training, she has to conduct an audit project. Her trainer (a senior clinician) tells her that the practice has not achieved many cancer care quality points in the new general practice contract introduced in the UK [19, 20]. The senior clinician admits that there is no formalized approach for regularly reviewing patients with cancer. He asks Kate to help the practice address this deficiency, thereby communicating his willingness to give her freedom to plan the change.
Kate reads about the Macmillan Gold Standards Framework [18] – a credible source of evidence. The framework consists of seven key areas of palliative care practice. The practice has lunchtime meetings, and Kate describes the framework to two of the partners, a salaried GP and the practice's nurse practitioner. They all agree that it would be a good idea to audit the practice by using the framework as a guide. During the training year, Kate and other practice members make changes to the way palliative patients are reviewed and their caregivers identified. The nurse practitioner purchases a whiteboard, which is completed, updated, and gives information about the entirety of ongoing terminal care cases. The out-of-hours emergency service is provided with details about the active caseload. Kate writes a report about the work and her trainer submits the project for a national competition of improvement projects in general practice.
A few months later, her work wins the first prize of £3000 and a £1000 award celebratory dinner for the entire practice. Whereas in the previous year, the practice scored poorly on cancer care quality points, in the following year, the maximum score is obtained.

### Communication theory and Knowledge transfer milestones

Stickiness is a product of the transfer process, and can be predicted by examining a number of conditions relating to the knowledge, its source, the context of the transfer, and the characteristics of the recipient.

**Figure 1:**
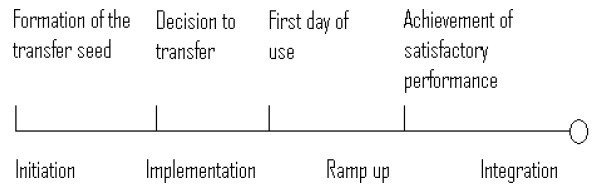
Knowledge transfer milestones [21].

Szulanski conceptualized the phases of knowledge transfer using the milestones described by Van de Ven [19], to examine stickiness during the process (see Figure 1).

The first milestone is named the transfer seed. This is early recognition that either a gap in knowledge or use of knowledge exists, or that someone discovers better knowledge or an improved way of doing things.

Let's take as an example the Gold Standards Palliative Framework [18]. The framework recognizes the need for a structured protocol for palliative care in a community setting, a framework that is sufficiently generic to fit most if not all practice contexts. Family doctors may recognize that their palliative care provision needs improvement, but may not know how to approach this systematically. This perception represents a knowledge gap. As in our example, this perception may be triggered by external forces, such as loss of quality points, and thereby potential loss of income.

This perceived knowledge gap acts as a trigger, a transfer seed for an organization to seek more information and to consider the second stage, a decision to transfer, or in the healthcare literature, a decision to implement. The second milestone is recognized by a decision process, often observed as a formal process such as the approach of a governing or decision-making body in the organisation or the signing of a contract. The empirical surrogate for this decision to transfer milestone is the beginning of recognizable activity such as the arrival of a person, documents, or machinery. Ordering an introduction pack, then sharing and agreeing (in a partnership meeting) to try out the Gold Standards Palliative Care Framework at Kate's practice represents a decision to transfer.

The third milestone is first day of use, where the knowledge is activated. Signs can include the physical switch to a new process, the abandonment of an old computer system, bringing a new plant on stream, switching personnel roles, etc. The fact that Kate is actively following the guidelines set out by the Gold Standards Framework by creating a list of patients who are terminally ill and reviewing their individual notes with the question "Have they had a cancer care review in the last three months?" is an illustration of this stage.

Achieving effective performance is the fourth milestone, and normally takes much more time as processes are ramped up to speed. Kate is setting up an audit to monitor the change she is implementing, and the results should demonstrate how well or how poorly the practice has performed. In addition, the process has added a system of tracking with a whiteboard, and the partners have agreed to check these patients out on the emergency call coverage.

These then are the milestones of knowledge transfer: formation of transfer seed, decision to transfer, first day of use, and the achievement of satisfactory performance. Szulanski sorted these further into four chronological stages: initiation and implementation, two stages that are characterized by learning before doing (planning and experimenting), followed by ramp-up and integration, two stages characterized by learning by doing (resolving problems, then follow-through and adaptation). Now, let us explore where things can go wrong, or stick in this knowledge transfer. In Case Study, Year Two, (Table 2) Kate has moved on to an inner city practice.

**Table 2:** Case Study, Year 2. An unreceptive environment and arduous relationships

Kate has finished her training year and is working as a 0.6 full-time equivalent salaried family doctor in a busy practice in central London. Brimming with enthusiasm after winning a prize for successfully implementing palliative care improvement in her previous practice, she decides to talk to the partners and the practice manager about instituting the Gold Standards Framework in this practice. It proves difficult to get all the relevant people to meet, as there are no informal meetings. There are two formal practice meetings a week but they have full agendas, and it proves difficult to add a new item. In addition, the meetings rarely achieve consensus. Kate decides to use the practice's e-mail system and sends a message to all the clinicians describing her proposal to address the quality of palliative care by using a proven method and framework of best practice. She only receives one reply, which although encouraging ends by saying "we already are doing enough for cancer, but we need to look at flu-vaccination uptake if that's of interest to you?". In addition, one of the senior doctors views Kate as lacking the necessary experience to introduce changes into their organization. Kate perseveres, but two months later has only managed to achieve four of the seven points set out by the Framework. She wants contributions from the clinicians to maintain and update the profiles of patients receiving palliative care, but has to resort to repeated prompting to obtain information, compared with her experience at her previous practice where this was done automatically and where clinical records were updated as part of routine practice. Kate feels unsupported and her motivation to continue implementing the framework wanes.

Szulanski proposed predictors of stickiness have different characteristics and importance during different stages of knowledge transfer. From this examination of the mechanics of transfer, Szulanski identified nine predictors of stickiness, see Table 3.

**Table 3:** Predictors of stickiness at different points of knowledge transfer

**Communication elements**	**Predictors of stickiness**
Knowledge	1. Causal ambiguity
	2. Unproven knowledge

Source	3. Motivation of source
	4. Credibility of source

Recipient	5. Recipient motivation
	6. Recipient absorptive capacity
	7. Recipient retentive capacity

Context	8. Barren organisational context
	9. Arduous relationship between source and recipient

### Causal ambiguity

Causal ambiguity exists where precise reasons for success or failure of knowledge transfer are unknown. The exact conditions of the best practice cannot be reproduced, and the impact of idiosyncrasies of the new environment cannot be fully understood [20]. This is a problem that is related to the gap between what should be done and what is actually done. Kate described how the new system would work at relaxed daily lunchtime meetings in her first practice, whereas she met overbooked, conflict-laden agendas at her next practice. The partners in the second practice had no conception of what should be done, and there was no opportunity for them to see how the system would or might work to their advantage. Szulanski describes this as know-why, and hypothesised that the greater the causal ambiguity the more difficult replication of best practice would be, and therefore the stickier the knowledge.

### Unproven Knowledge

Where the knowledge has a short, unproven track record or lack of evidence base, Szulanski reasoned it would arouse suspicion and therefore increase stickiness. Kate finds that in both practices no one has heard about the Gold Standards Framework and that it is a potential source of suspicion. At the second practice, Kate's lack of experience adds to the partners interpretation of the new idea being unproven, and it is therefore viewed with caution. This occurs despite the fact that, albeit relatively novel, Gold Standards Framework has already enjoyed success in primary care across the United Kingdom.

### Motivation of source

Stickiness, Szulanski hypothesised, was correlated with the motivation of the source to transfer it.

The cliché 'knowledge is power' resonates throughout industry and academia. Knowledge sharing and cooperation are unusual; competitiveness and using knowledge as currency for personal advantage are more common. Early reviewers of knowledge management suggest that sharing knowledge is an unnatural act [21]. This has been described as a culture of knowledge-hoarding [22]. For innovators to relinquish ownership of a best practice, they stand to lose control of its use, and this can lead to in an unwillingness to share [23]. It can result in covert sabotage of the transfer process, for example, withholding essential information or giving an incomplete description of the practice. This may not be pertinent to our example, as the source was clearly attempting to introduce best practice, but let us say that Kate had stayed at her first practice and that a neighbouring village practice had asked for assistance to introduce the same improvement. It would then depend very much on Kate's willingness to share her newly acquired knowledge.

### Credibility of source

Status and trustworthiness of the source may positively influence the ease of transfer. Szulanski notes that trustworthiness paradoxically may be a damaging to the transfer process, if the knowledge from a trustworthy source is flawed and the recipient assumes they do not have to critically appraise the delivered knowledge. On balance however, trustworthiness and credibility are likely to facilitate transfer, and therefore Szulanski hypothesised that lack of credibility in the source would be positively correlated with stickiness. It is difficult to say whether some family practices would find a newly qualified clinician credible. Obtaining professional qualifications such as the Membership of the Royal College of General Practitioners (MRCGP) could count positively towards Kate's credibility, but it is also likely that in some organisations credibility is linked more with time served and with loyalty to the *status quo *than to the introduction of innovations.

### Recipient motivation

Lack of motivation by a recipient to engage with new knowledge may be critical for the successful transfer of knowledge. The reluctance of recipients may manifest itself as foot-dragging, passivity, sabotage, fake acceptance, wilful rejection, and many more unattractive activities deployed by those seeking to maintain the *status quo *in the face of change. This lack of motivation is the most commonly cited reason for why efforts to transfer knowledge fail [24], and Szulanski also hypothesised that lack of motivation would be positively correlated with stickiness. In Kate's first practice, the nurse practitioner is so convinced by Kate's vision that she purchases a whiteboard for the cause. This in turn shows Kate that there are team members who are extremely motivated, perhaps due to their previous quality ratings on this topic, and will motivate her to persevere in introducing the new system. In the second case, the recipients do not appear to have concerns about the quality of their current palliative care and therefore little motivation for change.

### Recipient absorptive capacity

Related prior knowledge, existing skills, the ability to recognise value and seek sources of support for implementing a new practice, all add to the absorptive capacity of the recipient. A recipient lacking absorptive capacity is less likely to apply new knowledge successfully. This will increase costs, delay completion and may compromise the success of the transfer event. Therefore, if a recipient lacks absorptive capacity, Szulanski hypothesised stickiness would be increased. In Kate's second practice, numerous competing demands appear to decrease the providers absorptive capacity.

### Recipient retentive capacity

Transfer can be considered successful if there is long-term retention of the knowledge transferred, and the new practice is sustained in the participant's cognition. Sustainability is more likely where the new practice is used sufficiently to lose its novelty value and become embedded in routines. Retention is also more likely if old knowledge is destroyed or made unavailable so that it can't be reinstated. As an example, producing prominent laminated copies of the seven key areas of the Gold Standards Framework, and setting up automated computer reminders to review all cancer patients at least every four months helped to embed the new processes in the first example.

### Barren organisational context

Where innovations cannot get a toehold in organisations, the context could be said to be barren. Just like seeds, ideas, innovations, and new ways of doing things need protection and nourishment to survive. Where favourable conditions are not available, new practices cannot flourish. Barren organisational context was therefore identified as a predictor of stickiness. In Kate's second practice, none of the participants could imagine the new system and the advantages it would bring.

### Arduous relationship between source and recipient

Knowledge transfer is rarely an isolated event, but rather part of a continuing relationship between the source and recipient. As such, the relationship for transfer will be modified by past experience, including characteristics such as previous intimacy, ease of communication, support in the process, recognitions of success, and absence of penalties for failure. In our first example, the knowledge gap is admitted by a senior decision-maker, and Kate's enthusiasm is reciprocated by a supporting organisation. In the second practice, the relationship between Kate and the recipient organisation is much more difficult, and she is perceived as not being aligned with their own priorities. The more arduous the relationship, Szulanski hypothesised, the stickier the knowledge transfer process would become.

### Szulanski's findings

Szulanski's study sought to answer two questions, how does stickiness manifest itself at each point in the transfer process, and what are the best predictors of difficulty for each stage of the transfer?

The most surprising finding was that knowledge factors – causal ambiguity, absorptive capacity, and reliability – were significantly more important than motivation of the recipient [13]. This places the responsibility for successful change management with the organization, its management, structure, resourcing, and facilitation of the process. It releases individuals from being scapegoated (often by management) as unmotivated spoilers of reasonable requests to change behaviors.

### The sticky knowledge in transferring best practice

#### Initiation stickiness

This stickiness relates to difficulties in recognizing opportunities for transferring best practice and acting on them. Szulanski notes that recognizing the opportunity requires a significant investment of time and effort in delimiting and defining the best practice to be transferred, and then taking the initiative to decide when and how to begin the transfer process. Had the partners in Kate's second workplace been more prepared to listen and engage, they may have recognized potential advantages for themselves and their patients. Or if Kate had spent sufficient time to first understand what was currently been done for palliative care in the new practice, why there was or was not a perception of need to change, and what the competing priorities in the practice were, she might have had a more receptive audience when she did approach the leadership.

#### Implementation stickiness

During the phase when new knowledge is implemented, stickiness is related to the technical and communication gaps between the source and the recipient of the knowledge. Bridging this gap successfully is related to careful planning, however the depth of the planning is itself dependent on the understanding of the best practice being transferred, *i.e*., on the degree of causal ambiguity. How likely the effects of causal ambiguity are to derail the transfer process will be dependent on the ability of the source and recipient to work together to resolve conflicts, oversights, and misconceptions. Hence, stickiness during implementation is also dependent on the relationship between the source and the recipient. Kate, being the source of the new practice, would have to work at every step of the process and foresee potential pitfalls, both in terms of relationship building and avoiding technical problems.

#### Ramp-up stickiness

Causal ambiguity – when precise reasons for success are not really understood – is again implicated in stickiness during this stage. The greater the causal ambiguity of the best practice, the more likely it is that problems will be encountered during this phase when the newly transferred knowledge is implemented and performance is expected to exceed that of the previous practice. Problems are easier to resolve when the causal relationships are well-understood; whereas, when there is causal ambiguity there will be greater difficulty resolving problems associated with transfer.

#### Integration stickiness

If the new knowledge presents too many problems, it is unlikely to become part of everyday routine and therefore normalized (sustained) in practice. When difficulties are encountered, the new practice may be abandoned. In a recent qualitative study, family practitioners were reluctant to use the urea breath test for detection of Helicobacter pylori, as the test requires patient supervision and considerable clinical staff time [25]. Success here depends on ability to remove obstacles and deal with how to make the new practice more routine.

#### Sticky knowledge and improvement of health care quality and safety

Recent reviews of how to transfer best practice in health systems have not given definitive solutions [2,26], but they do lend support to Szulanski's findings that knowledge factors play a greater role in the success or failure of a knowledge transfer than has been suspected. Greenhalgh, for example, notes there is consistent empirical evidence to support absorptive capacity of recipient as a facilitator of transfer [26]. Kate's first surgery was a willing recipient. For those wishing to spread best practice, these findings promoting the importance of knowledge-related factors in transfer, if replicated, could have several practical implications.

#### Sticky knowledge is normal

The language of transfer and our preoccupation with why it doesn't work presupposes that it should be easy, and in a normal situation somehow transfer wouldn't be riddled with problems. Such a view is mistaken. Transfer is normally sticky. In the complex system we have been discussing there is no easy way to transfer, and the holy grail of change without effort simply doesn't exist. Szulanski is at pains to stress this, and his method is designed to embrace the problem as the norm, not the exception to the norm. He suggests social action is an effortful endeavour, and transfer requires endless problem-solving; he points to the work of Carlile, who states that normality is full of problems, difficulties, and failures, and that success can only be achieved through effort [27]. Nevertheless, we recognise that this approach seems very structured and categorical, whereas much of recent thinking has been about recognising the emergent, iterative, and adaptive manner in which evidence is understood [28] and change develops, suggesting that only certain aspects of any implementation remain under strategic control [29].

Change management, therefore, is not for the faint-hearted, or those lacking curiosity and creativity in their approach to problems. For busy clinicians and managers, attempting to get to terms with intangibles like knowledge capital may seem too much to take on when the to-do list is already full. However, for those who have tried and failed to transfer best practice, or those who are puzzled by the indifference their colleagues show towards evidence of best practice, sticky knowledge may play a role in helping overcome the barriers to transfer. By focusing our attention on how we move and manage knowledge in all its subtleties, we may find some of the answers we are looking for; the challenge of how to spread good ideas may be won with an armoury based on knowledge tools with an empirically tested evidence base.

## Competing interests

The author(s) declare that they have no competing interest.

## Authors' contributions

GE drafted the manuscript. GE, MT and JK participated equally in the writing of the article. All authors read and approved the final version.
